# Cardiac Wnt5a and Wnt11 promote fibrosis by the crosstalk of FZD5 and EGFR signaling under pressure overload

**DOI:** 10.1038/s41419-021-04152-2

**Published:** 2021-09-25

**Authors:** Yan Zou, Le Pan, Yi Shen, Xiang Wang, Chenxing Huang, Hao Wang, Xuejuan Jin, Chao Yin, Ying Wang, Jianguo Jia, Juying Qian, Yunzeng Zou, Hui Gong, Junbo Ge

**Affiliations:** Shanghai Institute of Cardiovascular Diseases, Zhongshan Hospital, and Institutes of Biomedical Sciences, Fudan University, Shanghai, 200032 China

**Keywords:** Heart failure, Translational research

## Abstract

Progressive cardiac fibrosis accelerates the development of heart failure. Here, we aimed to explore serum Wnt5a and Wnt11 levels in hypertension patients, the roles of Wnt5a and Wnt11 in cardiac fibrosis and potential mechanisms under pressure overload. The pressure overload mouse model was built by transverse aortic constriction (TAC). Cardiac fibrosis was analyzed by Masson’s staining. Serum Wnt5a or Wnt11 was elevated and associated with diastolic dysfunction in hypertension patients. TAC enhanced the expression and secretion of Wnt5a or Wnt11 from cardiomyocytes (CMs), cardiac fibroblasts (CFs), and cardiac microvascular endothelial cells (CMECs). Knockdown of Wnt5a and Wnt11 greatly improved cardiac fibrosis and function at 4 weeks after TAC. In vitro, shWnt5a or shWnt11 lentivirus transfection inhibited pro-fibrotic effects in CFs under mechanical stretch (MS). Similarly, conditional medium from stretched-CMs transfected with shWnt5a or shWnt11 lentivirus significantly suppressed the pro-fibrotic effects induced by conditional medium from stretched-CMs. These data suggested that CMs- or CFs-derived Wnt5a or Wnt11 showed a pro-fibrotic effect under pressure overload. In vitro, exogenous Wnt5a or Wnt11 activated ERK and p38 (fibrotic-related signaling) pathway, promoted the phosphorylation of EGFR, and increased the expression of Frizzled 5 (FZD5) in CFs. Inhibition or knockdown of EGFR greatly attenuated the increased FZD5, p-p38, and p-ERK levels, and the pro-fibrotic effect induced by Wnt5a or Wnt11 in CFs. Si-FZD5 transfection suppressed the increased p-EGFR level, and the fibrotic-related effects in CFs treated with Wnt5a or Wnt11. In conclusion, pressure overload enhances the secretion of Wnt5a or Wnt11 from CMs and CFs which promotes cardiac fibrosis by activation the crosstalk of FZD5 and EGFR. Thus, Wnt5a or Wnt11 may be a novel therapeutic target for the prevention of cardiac fibrosis under pressure overload.

## Introduction

Cardiac fibrosis, induced by a variety of cardiac injuries, reduces ventricle wall compliance, and disrupts cardiac conduction, leading to the decreased cardiac output and the development of heart failure. Progressive cardiac fibrosis is predominantly mediated by the activation of resident cardiac fibroblasts (CFs) in response to pressure overload or myocardial infarction (MI) injury [1, 2]. Inhibition of activation of resident CFs would be an attractive therapeutic approach in heart failure. Importantly, the regulatory mechanisms that cause excessive deposition of extracellular matrix from CFs under pathophysiologic conditions, have not been fully elucidated.

Several signaling pathways including wingless (Wnt) signaling are dysregulated in the process of pathophysiological cardiac remodeling. Wnt proteins bind to a receptor complex composed of a Frizzled (FZD) receptor and a low-density lipoprotein receptor-related protein 5/6 (LRP5/6), to initiate signaling in different intracellular responses, involving β-catenin (canonical Wnt pathway), Ca^2+^, or other second messengers (non-canonical Wnt pathways) [3, 4]. We recently observed cardiac-specific LRP6 overexpression suppressed cardiac fibrosis and inhibited the secretion and expression of Wnt5a and Wnt11 in CMs under pressure overload [5]. Wnt5a and Wnt11, two non-canonical Wnt ligands, are co-required to promote second heart field development by restraining Wnt/β-catenin signaling in mice [6]. Wnt5a is elevated in the serum and myocardium of heart failure patients and associated with cardiac dysfunction [7, 8]. It has been reported that Wnt5a is derived from fibroblasts or myofibroblasts [7, 9] and induces cardiac hypertrophy by Dapper-1 [10]. Wnt11 is activated by TGF-β1 to drive mesenchymal gene expression in renal epithelial cells [11]. However, few evidences indicate that Wnt5a or Wnt11 directly promotes cardiac fibrosis during chronic pressure overload.

Here, we detected serum Wnt5a and Wnt11 levels in hypertension patients, and built a chronic pressure overload mouse model to detect cardiac Wnt5a and Wnt11 expression at different time-points, and explore the roles of cardiac-derived Wnt5a and Wnt11 in cardiac fibrosis and potential mechanisms under pressure overload.

## Material and methods

### Human study

In our study, hypertensive patients and controls (aged 50–80 years) were recruited from Zhongshan Hospital, Fudan University (Shanghai, China), from January to May 2016. There were 33 control patients and 56 hypertensive patients (including patients with hypertension lower drug treatment) were enrolled in the study. Hypertension was diagnosed according to the 2013 European Society of Hypertension (ESH)/European Society of Cardiology (ESC) Hypertension diagnostic criteria), systolic blood pressure (SBP) ≥140 mmHg and/or diastolic blood pressure (DBP) ≥90mmg. Thirty-three control patients who were free of any diseases were included in the study after a health checkup. Exclusion criteria: history of acute MI within 1 month, acute cerebrovascular accident, acute heart failure, acute infection within 2 weeks, severe hepatic and renal insufficiency and failure, to complete the examination. This study was approved by the Ethics Committee of Zhongshan Hospital, Fudan University (B2015-074). Family members/patients were given written informed consent prior to study inclusion. The investigation is consistent with the principles outlined in the Helsinki Declaration.

Human dilated cardiomyopathy (DCM) left ventricular tissues were obtained from patients who underwent heart transplantation with a diagnosis of DCM at Zhongshan hospital, Fudan University. Control heart tissues were from cardiac donors whose hearts were rejected for transplant or organ donor networks. The human heart tissue samples used in this study were approved by Ethics Committee at Zhongshan Hospital, Fudan University (Approval No: B2014-084). Consent for research use of explanted or the donor heart tissues was obtained in all cases.

### Mice

C57BL/6 male mice were housed in the animal facilities of the Fudan University (Shanghai, China). For different time-points after TAC, 25 mice aged 8–10 weeks were randomly divided into five groups including sham group, TAC 3 days, 7 days, 2 weeks, 4 weeks group (*n* = 5/group). For the shRNA-AAV9 experiment, 22 mice aged 7–8 weeks were randomly injected with shRNA-scramble-AAV9 (sh-NC) or shRNA-Wnt5a/11-AAV9 (shWnt5a/11) (*n* = 11/group). Two weeks later, sh-NC or shWnt5a/11 mice were randomly subjected with TAC or sham operation (*n* = 5/sham group; *n* = 6/TAC group). There are one mice in shNC-TAC and shWnt5a/11-TAC groups died after TAC operation. For Wnt5a/11 infusion experiment, 24 mice aged 8–10 weeks were randomly divided into four groups (control, TAC, Wnt5a/11 infusion, TAC + Wnt5a/11 infusion, *n* = 6/group). An osmotic infusion was inserted into the back 2 days before TAC. No mice died after the TAC operation. All the animal experimental protocols were approved by the Animal Care and Ethics Committee of Zhongshan Hospital, Fudan University.

### Serum Wnt5a and Wnt11 levels analysis

The level of Wnt5a and Wnt11 in serum was determined by ELISA analysis. In brief, blood samples, which we collected from patients, were centrifuged with 1000x*g* at 4 ^°^C for 20 min, and then the supernatant was collected for analysis of Wnt5a and Wnt11 level by ELISA Kit (CUSABIO, Wuhan, China) according to the manufacturer’s protocols.

### Mechanical stretch

We used a stretching device to simulate the pressure overload model in vitro. Silicone sheets were pre-coated with 0.1% acetic acid rat tail collagen. Cells were cultured in these silicone sheets for 2–3 days and then deprived of serum for 24 h. For a stretch, the silicone sheet was fixed in the stretching frame which was put in a 150-mm culture dish. The uniaxial strain was provided by stretching the silicone sheet in the frame, and the silicon sheet was stretched to 120%. The control cells were also grown on the silicone sheet without giving stretch. At specific time-points, the cells or the culture medium were harvested for analysis.

### Thoracic aortic constriction (TAC) in mice

The pressure overload mouse model was built by TAC. C57BL/6 male mice aged 8–10 weeks were anesthetized and artificially ventilated, then the transverse aorta was ligated by 6-0 nylon suture together with a blunted 27-gauge needle, which was later pulled out. Sham-operated mice were also included whereby the same procedures were carried out except for aortic constriction. At the different time-points (3, 7, 14, and 28 days) after TAC or sham operation, further analysis were performed.

### Wnt5a and Wnt11 infusion

Eight–12 weeks C57BL/6 male mice were subjected to sham or TAC operation and then administered with Wnt5a (560 ng/kg/d) and Wnt11 (86 ng/kg/d) or vehicle (as control) continuously for 2 weeks by implanting subcutaneously Alzet osmotic minipumps (Model 1002; DURECT, Cupertino, CA, USA). Echocardiography or molecular analysis was performed at 4 weeks after sham or TAC operation.

### Echocardiography analysis

The echocardiography was obtained using VisualSonics Vevo2100 animal imager (VisualSonics, Inc.). The mice were anesthetized with isoflurane, to keep the heart rate (HR) at >400 bpm. The echocardiographic parameters, including ejection fraction (EF), fraction shortening (FS), and HR were measured from M-mode images obtained from short-axis view.

### Western bot analysis

Total proteins were extracted from cells or heart tissues and quantitated using the BCA protein assay. According to the molecular weight of target proteins, the protein samples were separated in SDS/PAGE (10% gel), and then transferred to PVDF membranes. After blocking with Western blocking buffer, the membranes were incubated overnight at 4 ^°^C with primary antibodies: Wnt5a (1:1000,GTX111187), Wnt11 (1:1000,GTX105971), (all Gene Tex, San Antonio,TX, U.S.A.), FZD5 (1:1000, ab96547), TGF-β1 (1:1000, ab92486), alpha smooth muscle actin (α-SMA) (1:1000, ab124964), (all Abcam, Cambridge, MA, USA), phosphorylated extracellular-regulated protein kinase (ERK) (1:2000, #4370), ERK (1:1000, #4695), p-p38 (1:1000, #9211), p38 (1:1000, #9212), p-EGFR (1:1000, #4407), EGFR (1:1000, #2646), p-Smad2/3 (1:1000,#8828) (all Cell Signaling Technology, Danvers, MA, USA), MMP2 (1:1000,10373-2-AP), MMP9 (1:1000,10375-2-AP), Col-1 (1:1000, 14695-1-AP), (all Proteintech, Chicago, IL, USA), then incubated with horseradish peroxidase (HRP)–conjugated secondary antibodies (1:5000) for 1 h at room temperature. After interaction with Pro-Light chemiluminescent detection kit (willgel Biotech Inc, Shanghai, China), the proteins of the membranes were detected using OmegaLum C Gel Imaging system (Aplegen Inc, USA).

### Masson’s trichrome staining

The tissues were fixed with neutral formaldehyde solution for 24 h, subsequently embedded in paraffin and sliced (4–5 μM). These slides were incubated in Masson compound staining solution for 5 min. After cleaned with 0.1% acetic acid working solution, and incubated in the phosphomolybdic-phosphotungstic acid solution for differentiation, these slides were stained with the bright green-blue staining solution. Cardiac fibrosis (blue) was analyzed by software-Image J (National Institutes of Health, Bethesda, USA).

### Adeno-associated virus 9 vector (AAV9) construction

SiRNA-sequences targeted to mouse Wnt5a (CCGGGCTAATTCTTGGTGGTC TCTACTCGAGTAGAGACCACCAAGAATTAGCTTTTTG) and Wnt11 (CCGGCCTGTGAAGGACTCAGAACTTCTCGAGAAGTTCTGAGTCCTTCACAGGTTTTTG) were screened out and sub-cloned into AAV9 plasmid for constructing AAV9-shWnt5a/11 plasmid to generate AAV9 particles by Hanheng Biotechnology Co., Ltd (Shanghai,China). Sh-Scramble was used as control. The AAV9 viruses 8.8 × 10^11^ particles were injected into tail vein of 6 weeks old mice. After 2–3 weeks, sham or TAC operation was performed.

### Neonatal CMs and CFs culture

We obtained the primary culture of CFs and CMs from 1–3 days old Sprague–Dawley (SD) rats by using the trypsin digestion method: the hearts were cut off quickly from neonatal rats and then washed twice with ice-cold PBS. Next, minced the heart into pieces and subjected to 0.25% trypsin digestion in Hank’s balanced salt solution digest 5–7 times. After 2 h of cell attachment, supernatant CMs and adherent CFs were collected and cultured with DMEM/F12, containing 10% FBS and 1% antibiotics for 24 h, 5-Bromo-2-deoxyuridine (5-BrdU, 0.1 mmol/L) was added into cultured CMs for 24 h to further purify CMs. α-MHC (one of the CM markers) immunostaining was performed to indicate the purity of CMs. The neonatal adherent cardiac fibroblast were treated with different concentrations of recombinant rat Wnt5a or Wnt11 from 0 to 100 ng/ml after incubation of serum-free medium for 24 h or subjected to MS to perform further analysis. Fibroblasts of second or third passage were used all throughout the present study for siRNA-epidermal growth factor receptor (EGFR) or frizzled 5 (FZD5) transfection experiments. Cells were cultured in serum-free DMEM medium overnight before treating with recombinant rat Wnt5a, Wnt11, or vehicle for indicated time periods. For the EGFR inhibitor study, fibroblasts were treated with Erlotinib (2 μM) or DMSO 1 h before treating recombinant Wnt5a, Wnt11, or vehicle. After 24 or 48 h, cells were harvested for the following experiments.

### CMECs culture

One-week-old male SD rats were anesthetized with 100 g /L chloral hydrate and fixed. Their hearts were removed quickly and rinsed with PBS. The right ventricle, atrium, large blood vessels were cut off, and the endocardium and epicardium should be removed as far as possible. The remaining heart tissue was cut into small tissue blocks of 1 mm^3^. The tissue mass was evenly coated into a 10 cm dish with 1 ml fetal bovine serum. The dishes containing tissue blocks were incubated in a 5% CO_2_ incubator until the tissue blocks firmly adhere to the dishes (about 4 h). And 6 ml DMEM containing 10% fetal bovine serum was added. After 48 h, the medium was exchanged. After 6 h, tissue blocks in the dishes were rinsed as far as possible with PBS, and 6 ml DMEM containing 10% FBS were added. When the degree of cell fusion reached 80%, 0.25% trypsin was used for cell passage.

### Immunofluorescence staining

The slides were placed in a wet box and washed with 1X PBS three times (5 min each time). 3% TritonX-100 permeable membrane 20 min, 1X PBS was washed three times. 5% BSA closed 1 h, α-MHC antibody (1:100; ab224046 from Abcam) was incubated overnight, 1X PBS was washed three times. The second antibody (1:200) was incubated at room temperature for 1 h,1X PBS was washed three times, Incubate DAPI at room temperature for 5 min1X PBS was washed three times. The anti-fluorescence quenching sealing tablets were sealed and observed under the fluorescence microscope.

### Fluorescence-activated cell sorting (FACS) analysis

#### Flow separation of negative and positive cells

The cultured fibroblasts were digested with trypsin, and the digestion was terminated with a termination solution. The cells were centrifuged at 1000 rpm for 5 min, and the supernatant was discarded. The cells were rinsed with PBS and centrifuged twice. α-SMA flow antibody (BS-0189R-APC, BIOSS) was diluted at a rate of 1:50, added into cells, and let stand at room temperature for 30 min. The cells were rinsed twice with PBS. After resuspending the cells with 1 ml PBS, α-SMA positive and negative cells were separated by a flow sorter (BD FACS Aira III).

### Lentivirus construction

SiRNA-sequences targeted to rat Wnt5a (5′-CCACGCCAAGGGCUCCUAUTT-3′) and Wnt11 (5′-CCACCAUCAGUCACACCAUTT-3′) were screened out and then construct lentivirus by Hanheng Biotechnology Co., Ltd (Shanghai, China). CMs or fibroblasts were evenly spread in a 6-well plate and cultured for 1–2 days. The original medium was replaced with a 2 ml fresh medium containing 6 μg/ml polybrene, and an appropriate amount of virus suspension was added for further incubation for 24 h. The medium containing virus was replaced with a fresh medium and the cells were collected 72 h after the addition of the virus.

### SiRNA transfection

The siRNAs targeted to rat EGFR (numbers 1166, 2258, and 3411) and rat FZD5 (numbers 444, 2944, and 1570) were designed and synthesized by GenePharma (Shanghai, China). siPORT^TM^ NeoFX^TM^ Transfection Agent (Ambion Inc, Texas, USA) was used for the transfection of EGFR and FZD5 siRNA according to the manufacturer’s protocols. Briefly, the EGFR and FZD5 siRNA (100 nM in 100 μl opti-DMEM) and transfection agent (5 in 100 μl opti-DMEM) were mixed and kept still for 10 min at room temperature, and then the mixtures (200 μl) were added into cultured fibroblasts.

### Real-time PCR (RT-PCR) analysis

Total RNA was extracted from fresh hearts, using UNlQ-10 Column Trizol Total RNA Isolation Kit(Cat#: B511321, Sangon Biotech, China), The concentrations of RNA samples were determined using Nano2000, the A260/280 was between 1.8 and 2.2. One microgram of total RNA was reverse transcribed into cDNA using the SYBR Premix ExTaq kit (Cat#: RR420A, Takara, Japan). The primers were synthesized by Tsingke Biotechnology (Shanghai, China). The specific PCR conditions were listed: Denaturation temperature 95 °C for 30 sec; annealing temperature 55 °C for 40 s; Extension temperature 72 °C, duration 60 sec, repeats 40. The results were analysed using 2^−ΔΔCt^ method. The primer sequences for RT-PCR were listed in Table S1.

### Statistical analysis

According to our preliminary experiments, for Wnt5a analysis, group sample sizes of 17 and 34 achieve 85% power to reject the null hypothesis of equal means when the population mean difference (effect size) is μ1 – μ2 = 0.2 − 0.1 = 0.1 with a standard deviation for both groups of 0.1 and with a significance level (alpha) of 0.050 using a two-sided two-sample equal-variance *t*-test; for Wnt11 analysis, group sample sizes of 26 and 52 achieve 85% power to reject the null hypothesis of equal means when the population mean difference (effect size) is μ1 – μ2 = 52.0 – 12.0 = 40.0 with a standard deviation for both groups of 50.0 and with a significance level (alpha) of 0.050 using a two-sided two-sample equal-variance *t*-test. Bonferroni adjusted for two pairwise comparisons. We used the larger sample size, finally included 33 controls and 56 HBP.

For human study, normal distribution variables were expressed as mean ± standard deviation (SD). The categorical variables comparison between groups was expressed as a percentage and analyzed by chi-square test. When the variables fit the normal distribution, the difference between the two groups was tested by unpaired two-sided Student’s *t*-test, in case two groups with unequal variances, Welch–Satterthwaite *t*-test were used; and Mann–Whitney *U*-test were used for skewed continuous variables. For correlation coefficient, Pearson correlation coefficient was selected for testing. For animal or cell research, data were expressed as mean ± standard error (SEM). The student’s *t*-test (unpaired, two-sided) was performed for two group comparisons. One-way ANOVA with Bonferroni post hoc test was used for multiple group comparisons. For analysis of shRNA-Wnt5a/11 or siFZD5 results, two-way ANOVA, followed by Bonferroni post-tests, was performed to evaluate the differences in mean values. For each animal experiment, C57BL/6 male mice with a similar age were randomly assigned to different experimental groups. The sample sizes including animal samples and cell experiments were determined according to our previous study and described in the figure legends. Investigators were not blinded by all these analysis. GraphPad Prism Version 8.0.2 and SPSS 26.0 statistical software were used to perform cartogram and statistical analysis. *P* value *<*0.05 was considered statistically significant.

## Results

### The levels of Wnt5a and Wnt11 are increased in serum of patients with hypertension

**Fig. 1:**
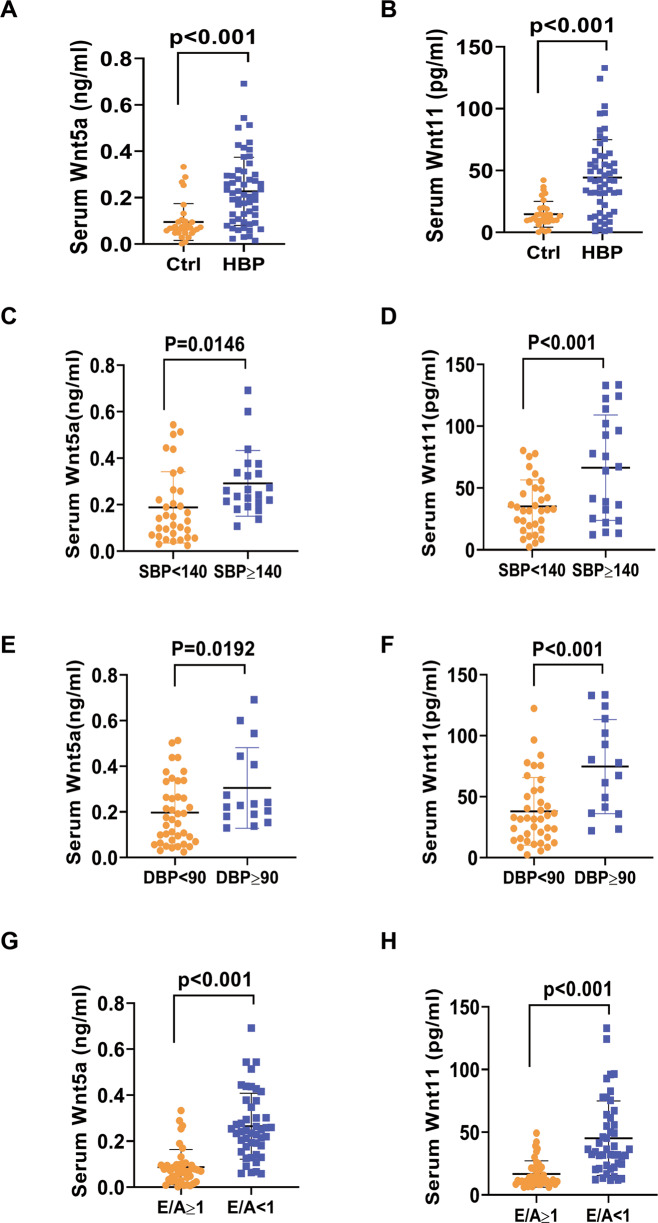
The levels of Wnt5a and Wnt11 are increased in the serum of patients with hypertension. The level of serum Wnt5a or Wnt11 was measured by ELISA analysis. **A**, **B** The level of serum Wnt5a or Wnt11 in hypertension group (HBP, *n* = 56) and control group (Ctrl, *n* = 33); **C**, **D** The level of serum Wnt5a or Wnt11 in SBP ≥ 140 mmhg group (*n* = 22) and SBP < 140 mmhg group (*n* = 34); **E**, **F** The level of serum Wnt5a or Wnt11 in DBP ≥ 90 mmhg group (*n* = 16) and DBP < 90 mmhg group (DBP, *n* = 40); **G**, **H** The E/A ratio between the healthy control group (Ctrl) and the hypertension (HBP) group was detected by echocardiography, and the ratio was divided into E/A ≥ 1 (*n* = 43) and E/A < 1 (*n* = 46). The level of serum Wnt5a or Wnt11 in the two groups were analyzed. SBP systolic blood pressure, DBP diastolic blood pressure. All data were expressed as means ± SEM.

Serum Wnt5a and Wnt11 levels were examined in patients with hypertension (HBP) and the control group. The basic characteristics of the two groups were shown in Table S2. SBP and DBP were higher in HBP groups than in the control group (SBP:137 ± 18 mmHg vs 127 ± 11 mmHg, *p* = 0.012; DBP:81 ± 10 mmHg vs 74 ± 9 mmHg, *p* = 0.001; Table S2). There is no obvious difference in the ratio of smokers and diabetes between the control and HBP groups. Hypertensive patients had the increased E (cm/s) value (76.68 ± 16.66 cm/s vs 70.05 ± 15.56 cm/s, *p* = 0.047) and E/e′ ratio (13.31 ± 6.73 vs 8.41 ± 2.88, *p* < 0.001), and the decreased e′ (cm/s) value (6.85 ± 2.85 cm/s vs 8.87 ± 2.47 cm/s, *p* = 0.002) compared with control group. There is no obvious difference in other parameters between the two groups (Table S2). ELISA analysis revealed higher Wnt5a and Wnt11 levels in hypertension patients than those in the control group (Wnt5a: 0.23 ± 0.15 ng/ml vs 0.11 ± 0.08 ng/ml, *p* < 0.001; Wnt11:52.34 ± 47.11 pg/ml vs 12.85 ± 10.54 pg/ml, *p* < 0.001, Fig. 1A, B). We divided the hypertensive participants into group SBP ≥140 mmHg and SBP <140 mmHg or DBP ≥90 mmHg and DBP <90 mmHg; Serum Wnt5a or Wnt11 was higher in group SBP ≥140 mmHg or DBP ≥90 mmHg than in group SBP <140 mmHg or DBP <90 mmHg, respectively (Fig. 1C–F). We also divided the participants into group E/A ≥ 1 and group E/A < 1. Serum Wnt5a or Wnt11 was higher in group E/A < 1 than in group E/A ≥ 1 (Fig. 1G, H). We then analyzed the relationship between serum Wnt5a or Wnt11 level and echocardiographic parameters of hypertension patients and the control group. Serum Wnt5a or Wnt11 level was positively correlated with E/e′ (Wnt5a: *r* = 0.273, *p* = 0.009; Wnt11: *r* = 0.32, *p* = 0.003), but not other parameters including LVEDD, EF, Septal thickness in the two groups (Table S3). We excluded the factors about smoke and diabetes and found serum Wnt5a and Wnt11 levels positively correlated to E/e′ (Wnt5a: *r* = 0.455, *p* = 0.010; Wnt11: *r* = 0.480, *p* = 0.006; Table S4). These data suggested that elevated serum Wnt5a and Wnt11 levels may be associated with diastolic dysfunction in hypertension patients.

**Fig. 2:**
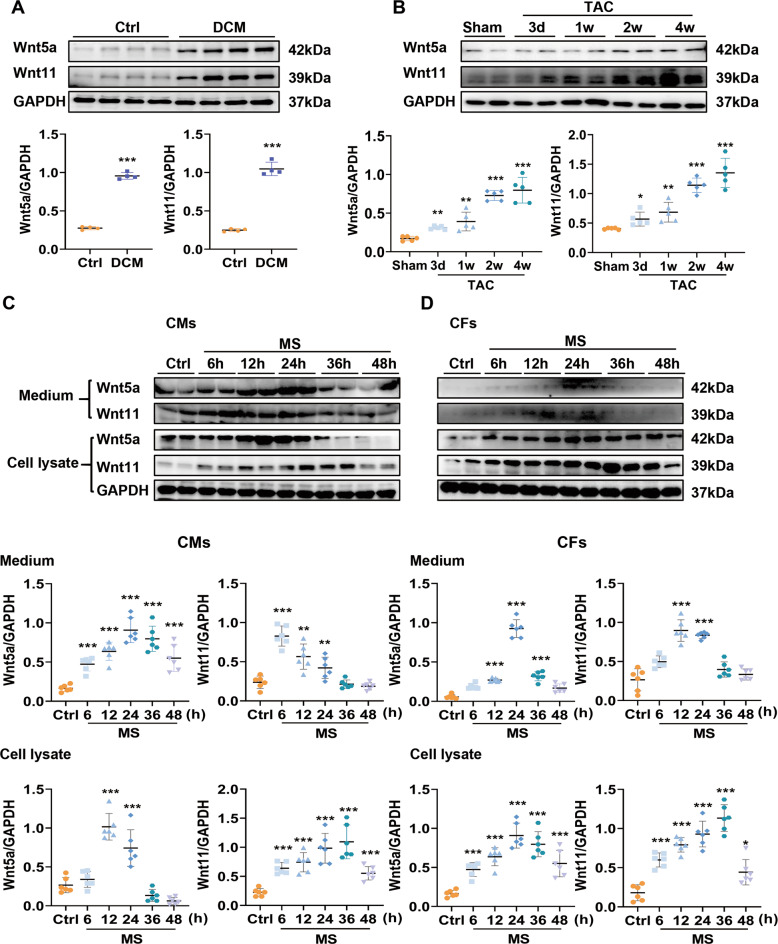
Pressure overload induces the increased expression and secretion of Wnt5a and Wnt11 from cardiomyocytes and cardiac fibroblasts. **A** The expressions of Wnt5a and Wnt11 were analyzed by western blot in the heart tissues of patients with dilated cardiomyopathy (DCM) or control (Ctrl) group, *n* = 4/group. **B** Western blot analysis of Wnt5a and Wnt11 expressions in heart tissues from TAC mice at different time-points (3 days, 1 week, 2 weeks, 4 weeks), *n* = 5/group. **C**, **D** Western blot analysis of Wnt5a and Wnt11 expression in cardiac myocytes (CMs), fibroblasts (CFs), and their supernatants at different time-points (6, 12, 24, 36, and 48 h) after mechanical stretch (MS), *n* = 6/group. All data were shown as mean ± SEM; *P < 0.05, **P < 0.01, ***P < 0.001.

We also analyzed the expression of Wnt5a and Wnt11 in heart tissue from DCM patients and the control group. As expected, fibrotic-related proteins Col-1, α-SMA, MMP2, MMP9, and TGF-β1 were higher in DCM hearts than in the control group (Fig. S1A). Cardiac Wnt5a and Wnt11 expression were sharply increased in DCM hearts compared to the control group (Fig. 2A). These data suggest that the increased cardiac Wnt5a and Wnt11 may be associated with cardiac remodeling induced by hypertension or other stress.

### Pressure overload induces the increased expression and secretion of cardiac Wnt5a and Wnt11

To explore the levels of cardiac Wnt5a and Wnt11 under pressure overload, we first examined cardiac Wnt5a and Wnt11 expression in different time-points (3 days, 1 week, 2 weeks, and 4 weeks) after TAC (Fig. S1B). Wnt5a and Wnt11 were both increased from 3 days and become higher in a time-dependent manner compared with the sham group (Fig. 2B), and the fibrotic-related markers including Col-1, MMP2, MMP9, and TGF-β1 showed the similar expression profile at different time-points after TAC (Fig. S1C). In vitro, we detected the expression in cultured neonatal rat CMs and CFs, and CMECs under MS respectively. α-MHC (one of the CM markers) immunostaining indicated the purity of CMs was about 93% (Fig. S2A). In basic conditions, the expression of Wnt5a or Wnt11 in CMs was higher than in CFs (Fig. S2B). CMs or CFs were subjected to MS at different time-points (Fig. S2C). In condition medium of CMs, the level of Wnt5a and was increased from 6 h and maintained high level until 36 h after stretch (Fig. 2C). CM-expressed Wnt5a and Wnt11 were both higher at 12 h and 24 h after MS than those in the control group (Fig. 2C). In a conditional medium of CFs, Wnt5a and Wnt11 were increased at 12–36 h after MS, compared to the control group (Fig. 2D). Fibroblast-expressed Wnt5a and Wnt11 began to increase from 6 h and kept high level until 48 h and 36 h, respectively, after MS, compared with the control group (Fig. 2D). α-SMA positive fibroblasts (sorted by FACS) expressed more Wnt5a or Wnt11 levels than α-SMA negative fibroblasts (Fig. S2D). Except for CMs or CFs, stretched-CMECs expressed more Wnt5a or Wnt11 than the control group (Fig. S2E).

These data indicated that the pressure overload induces the increased expression of Wnt5a or Wnt11 from CMs, CFs, and CMECs, which may be involved in the process of cardiac remodeling.

### Wnt5a and Wnt11 contributes to cardiac remodeling induced by pressure overload

**Fig. 3:**
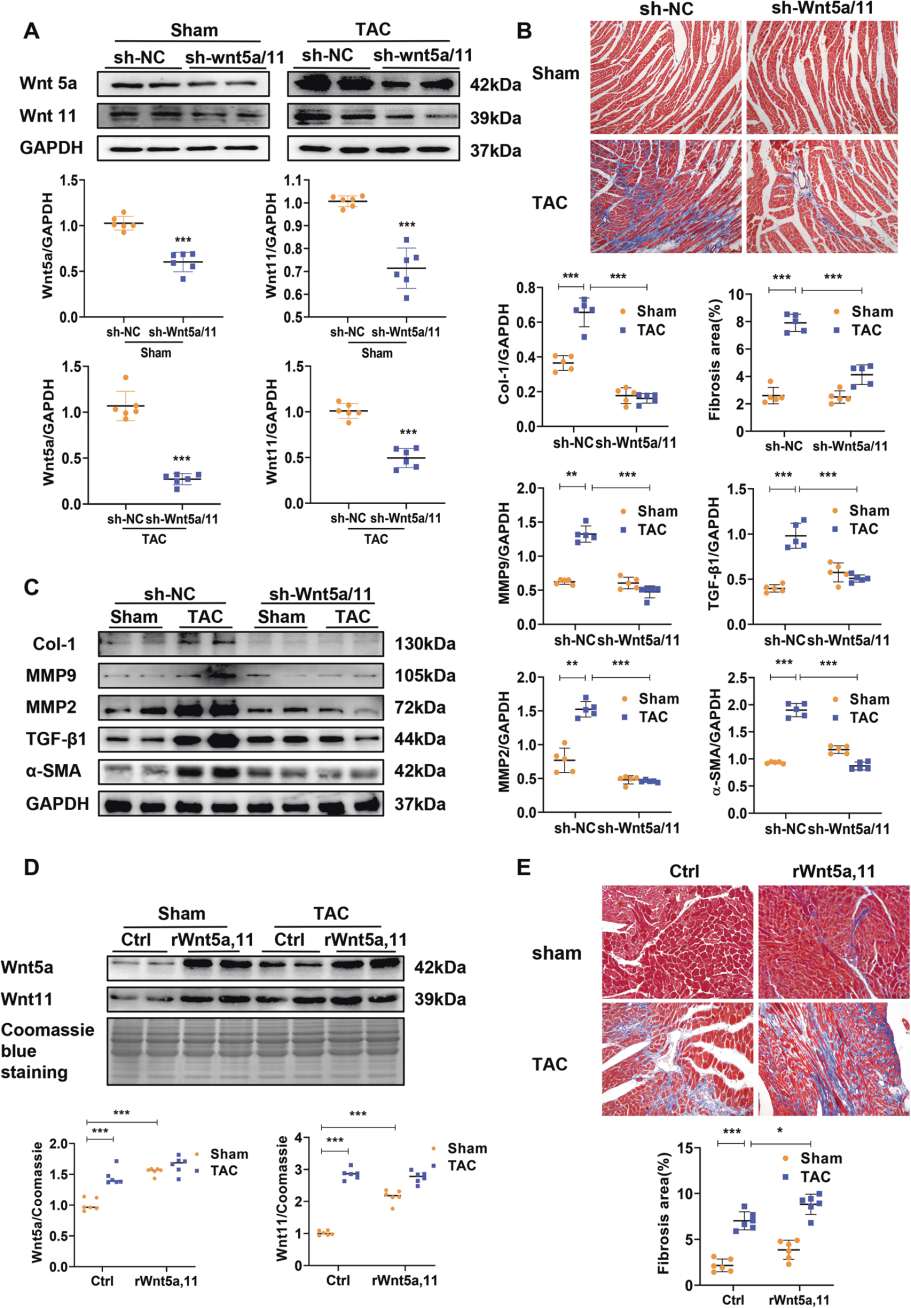
Wnt5a and Wnt11 contributes to cardiac remodeling induced by pressure overload. **A** Western blot analysis of Wnt5a and Wnt11 in heart tissues (*n* = 6/group). **B** Cardiac fibrosis was analyzed by Masson staining in heart tissues (*n* = 5/group). **C** Western blot analysis of Col-1, MMP2, MMP9, and TGF-β1 levels in heart tissues (*n* = 5/group). Mice were injected with sh-Wnt5A /Wnt11-AAV9 (shWnt5a/11) or sh-Scramble-AAV9 (sh-NC) by tail vein, 2 weeks later, these mice were subjected to TAC or sham operation. After 4 weeks, further analysis was performed. **D** Western blot analysis of serum Wnt5a or Wnt11 level at 4 weeks after sham or TAC operation, and these mice were infused recombinant rat Wnt5a (rWnt5a) and Wnt11 (rWnt11), or vehicle (as control) by minipump for 2 weeks from the sham or TAC operation (*n* = 6/group). **E** Cardiac fibrosis was analyzed by Masson staining in heart tissues (*n* = 6/group). All data were shown as mean ± SEM; *P < 0.05, **P < 0.01, ***P < 0.001.

shRNA-Wnt5a/11-AAV9 were injected into mice by tail vein to knock down the expression of cardiac Wnt5a and Wnt11. These mice were then subjected to TAC or sham operation. Wnt5a or Wnt11 was dramatically decreased in Sham and TAC mice pre-injected with shRNA-Wnt5a/11-AAV9 (Fig. 3A). Other Wnts mRNAs such as Wnt1, Wnt4, Wnt7a, and Wnt10b did not alter in TAC hearts by shRNA-Wnt5a/11-AAV9 injection (Fig. S3A). TAC induced obvious cardiac fibrosis, and the decreased FS, and shRNA-Wnt5a/11-AAV9 pre-injection greatly improved the effects after TAC (Fig. 3B and Fig. S3B). TAC induced the increased expression of fibrotic-related proteins, Col-1, α-SMA, MMP9, MMP2, and TGF-β1, were significantly inhibited by shWnt5a/11-AAV9 treatment (Fig. 3C). In addition, HW/BW ratio, cross section area (CSA) of CMs, mRNA levels of ANP, and BNP (hypertrophic genes), showed similar results (Fig. S3C–E).

We infused Wnt5a and Wnt11 into sham or TAC mice for 2 weeks, and observed serum Wnt5a or Wnt11 level was significantly increased at 4 weeks after sham or TAC operation compared with their control group, respectively (Fig. 3D). Wnt5a and Wnt11 infusion induced a slight increase in fibrosis area and the expression of fibrotic-related proteins (Col-1, MMP2, and MMP9) in sham mice, and exaggerated cardiac fibrosis in TAC mice (Fig. 3E and Fig. S3F). HW/BW ratio showed a similar effect in TAC mice (Fig. S3G). But the infusion did not deteriorate the decreased FS in TAC mice (Fig. S3H).

These data suggested elevated Wnt5a or Wnt11 contributes to cardiac hypertrophy and fibrosis under pressure overload.

### Cardiomyocyte-secretory Wnt5a or Wnt11 is involved in pro-fibrotic effects under MS

**Fig. 4:**
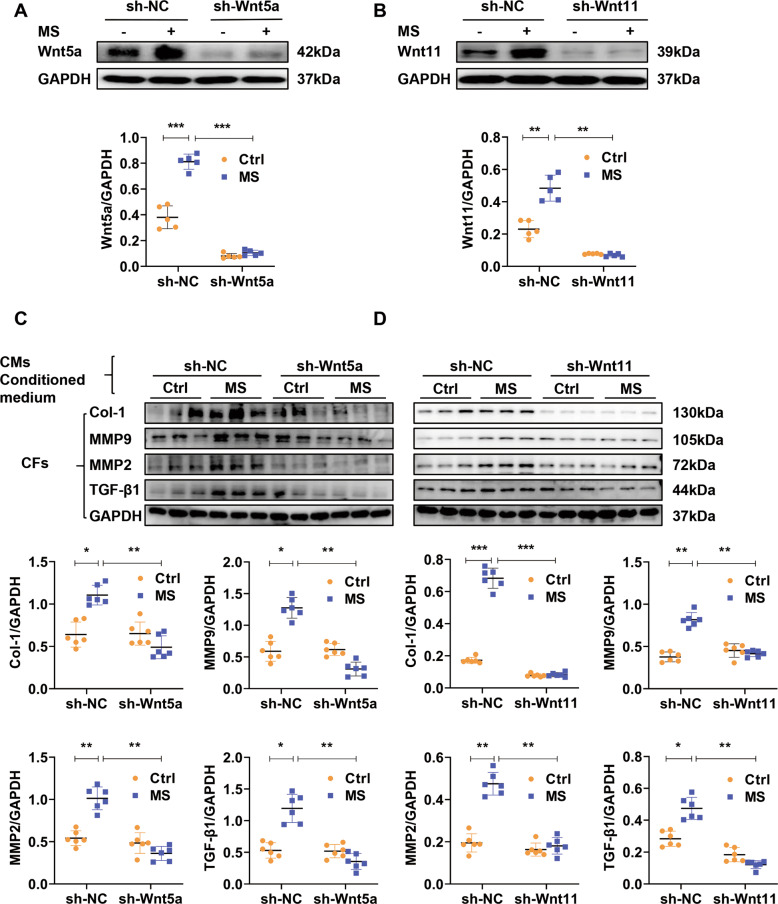
Cardiomyocyte-secretory Wnt5a or Wnt11 is involved in pro-fibrotic effects under pressure overload. **A**, **B** Western analysis of Wnt5a or Wnt11 in stretched (MS) or control (Ctrl) cardiomyocytes pre-transfected sh-Scramble (sh-NC), sh-Wnt5a or sh-Wnt11 lentivirus, respectively. *n* = 5/group. **C**, **D** Western analysis of Col-1, MMP9, MMP2, and TGF-β1 in cardiac fibroblasts (CFs) treated with conditional medium from cardiomyocytes (CMs) in (**A**) or (**B**), respectively. *n* = 6/group Stretched (MS) or control (Ctrl) cardiomyocytes were pre-transfected with sh-Scramble (sh-NC), ()shWnt5a or shWnt11 lentivirus, respectively. The conditional medium of these cardiac myocytes was collected to treat fibroblasts for 24 h, and the expressions of Col-1, MMP9, MMP2, and TGF-β1 were detected by Western blot. All data were shown as mean ± SEM; *P < 0.05, **P < 0.01, ***P < 0.001.

To study whether CM-secreted-Wnt5a or -Wnt11 is involved in the cardiac fibrosis under pressure overload, we knocked down Wnt5a or Wnt11 by pre-transfection of shRNA-Wnt5a or shRNA-Wnt11 lentivirus (shWnt5a or shWnt11) into CMs. Wnt5a or Wnt11 was sharply decreased by shWnt5a or shWnt11 in cultured CMs with or without MS (Fig. 4A, B). The conditional medium of these CMs was provided into CFs. The expression of Col-1, α-SMA, MMP9, MMP2, and TGF-β1, were greatly enhanced in fibroblasts by conditional medium from stretched-CMs, and the effects were significantly attenuated by the conditional medium from stretched-CMs pretreated with shWnt5a or shWnt11. There were no obvious differences in these fibrotic-related markers expression in fibroblasts treated with conditional medium from shRNA-Wnt5a or shRNA-Wnt11 and shRNA-scramble of the control group (Fig. 4C, D).

These data suggested that CM-secretory Wnt5a or Wnt11 is involved in pro-fibrotic effects under pressure overload.

### Fibroblast-expressed Wnt5a or Wnt11 is involved in pro-fibrotic effects under MS

**Fig. 5:**
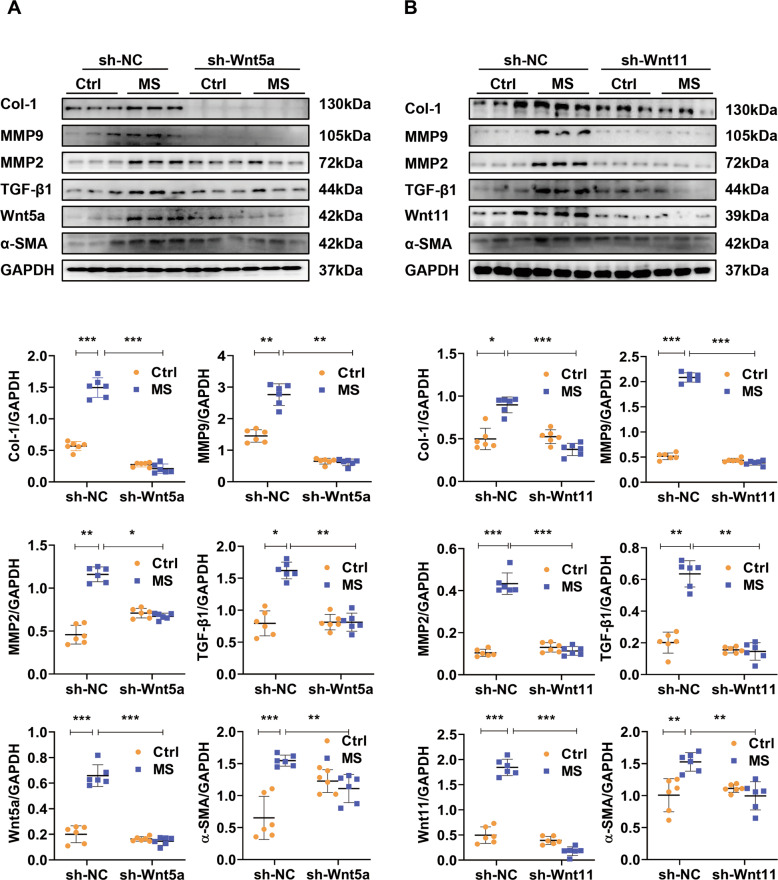
Fibroblast-expressed Wnt5a or Wnt11 is involved in pro-fibrotic effects under pressure overload. **A**, **B** Western analysis of Wnt5a, Wnt11, Col-1, α-SMA, MMP9, MMP2, and TGF-β1 in stretched (MS) or control (Ctrl) cardiac fibroblasts pre-transfected with sh-Wnt5a, sh-Wnt11 or sh-scramble (sh-NC) lentivirus, respectively. *n* = 6/group. All data were shown as mean ± SEM; *P < 0.05, **P < 0.01, ***P < 0.001.

We then detected the effect of fibroblast-expressed Wnt5a or Wnt11 in cardiac fibrosis under MS. CFs were pre-transfected with shRNA-Wnt5a or shRNA-Wnt11 lentivirus (sh-Wnt5a or sh-Wnt11) and subjected to MS. Sh-Wnt5a or sh-Wnt11 effectively downregulated the expression of Wnt5a or Wnt11 in fibroblasts with or without MS (Fig. 5A, B). Stretch induced the increased expression of Col-1, α-SMA, MMP9, MMP2, and TGF-β1, compared with the control group, sh-Wnt5a or sh-Wnt11 pre-transfection significantly inhibited the fibrotic effects in stretched-fibroblasts (Fig. 5A, B).

These data indicated that fibroblast-expressed Wnt5a or Wnt11 induces pro-fibrotic effects under pressure overload.

### Exogenous rat Wnt5a or Wnt11 activates fibrosis-related signaling in neonatal rat CFs

**Fig. 6:**
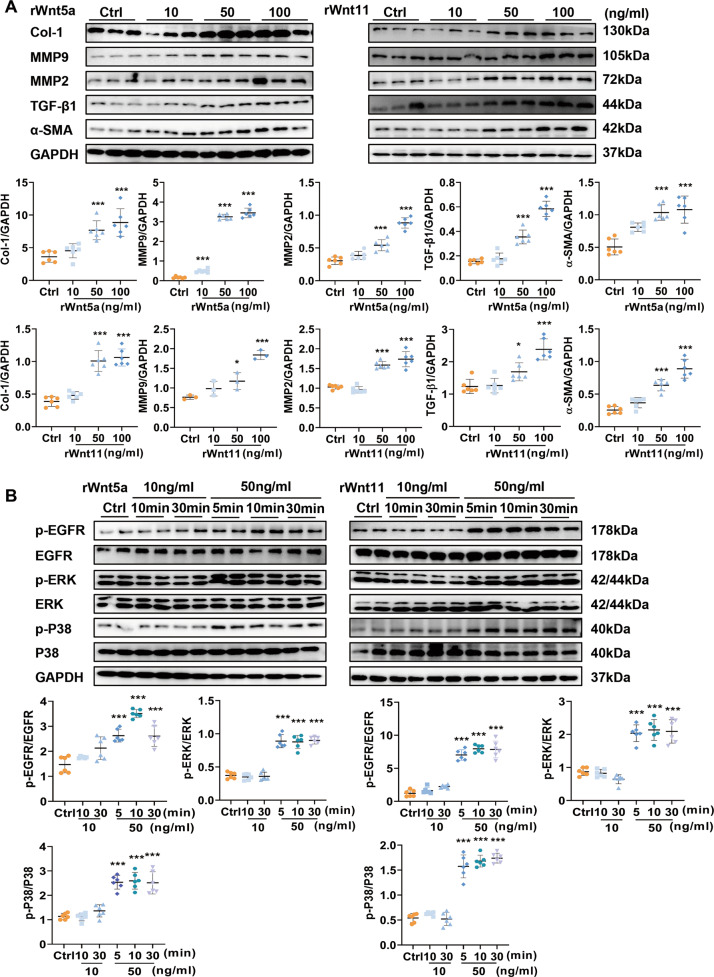
Exogenous Wnt5a and Wnt11 activates fibrosis-related signaling in neonatal rat cardiac fibroblasts. **A** The expression of Col-1, MMP9, MMP2, and TGF-β1 were detected by western blot analysis in cardiac fibroblasts stimulated with exogenous recombinant rat Wnt5a (rWnt5a) or Wnt11 (rWnt11) at different concentrations (0, 10, 50, 100 ng/ml) for 24 h. *n* = 6/group. **B** The levels of p-EGFR, p-ERK, and p-P38 were examined in cardiac fibroblasts stimulated with exogenous recombinant rat Wnt5a (rWnt5a) or Wnt11 (rWnt11) at different concentrations (0, 10, 50, 100 ng/ml) and different time-points (0, 5, 10, 30 min), *n* = 6/group. All data were shown as mean ± SEM; *P < 0.05, **P < 0.01, ***P < 0.001.

We treated cultured neonatal rat CFs with exogenous rat Wnt5a or Wnt11 at different concentrations (10, 50, 100 ng/ml). The fibrotic-related proteins, Col-1, α-SMA, MMP2, MMP9, and TGF-β1 were increased in neonatal rat CFs at 24 h after Wnt5a or Wnt11 in a concentration-dependent manner (Fig. 6A). Wnt5a or Wnt11 (both 50 ng/ml) showed similar results in cultured CFs at 24 and 36 h (Fig. 4SA, B). These data suggested that exogenous rat Wnt5a or Wnt11 continuously activated CFs. In addition, we also found Wnt5a or Wnt11 activated ERK signaling (Fig. S4C) suggesting a critical role in hypertrophic response in cultured CMs.

P38 and ERK pathway and EGFR activation are involved in the development of cardiac fibrosis [12]. Wnt5a or Wnt11 significantly induced the increased p-P38, p-ERK level, and p-EGFR in cultured CFs in a time or concentration-dependent manner. (Fig. 6B). In vivo, p-EGFR was increased in the left ventricle after TAC, and the effect was suppressed by shWnt5a/11-AAV9 pre-injection (Fig. S5A).

Moreover, we also compared Wnt5a or Wnt11 level in conditional medium of neonatal rat CFs stimulated with Wnt5a, Wnt11, or MS. Wnt5a or Wnt11 level was higher at 24 h after MS than in Wnt5a or Wnt11 treatment group (Fig. S5B), suggesting MS-induces continuous expression or secretion of Wnt5a or Wnt11 in cultured CFs, which plays a critical effect in cardiac fibrosis.

These data suggested that pressure overload-induced continuous secretory of Wnt5a or Wnt11 from fibroblasts which may promote cardiac fibrosis by activation of EGFR, p38, and ERK signaling.

### Inhibition of EGFR activation attenuates the pro-fibrotic effect induced by Wnt5a or Wnt11 in neonatal rat CFs

We pretreated CFs with EGFR inhibitor, erlotinib, to observe the activation of fibroblast in the presence of Wnt5a or Wnt11. Erlotinib significantly inhibited the p-EGFR level and suppressed the increased expression of Col-1, α-SMA, MMP9, MMP2, and TGF-β1 induced in fibroblasts by Wnt5a or Wnt11 (Fig. S5C, D).

**Fig. 7:**
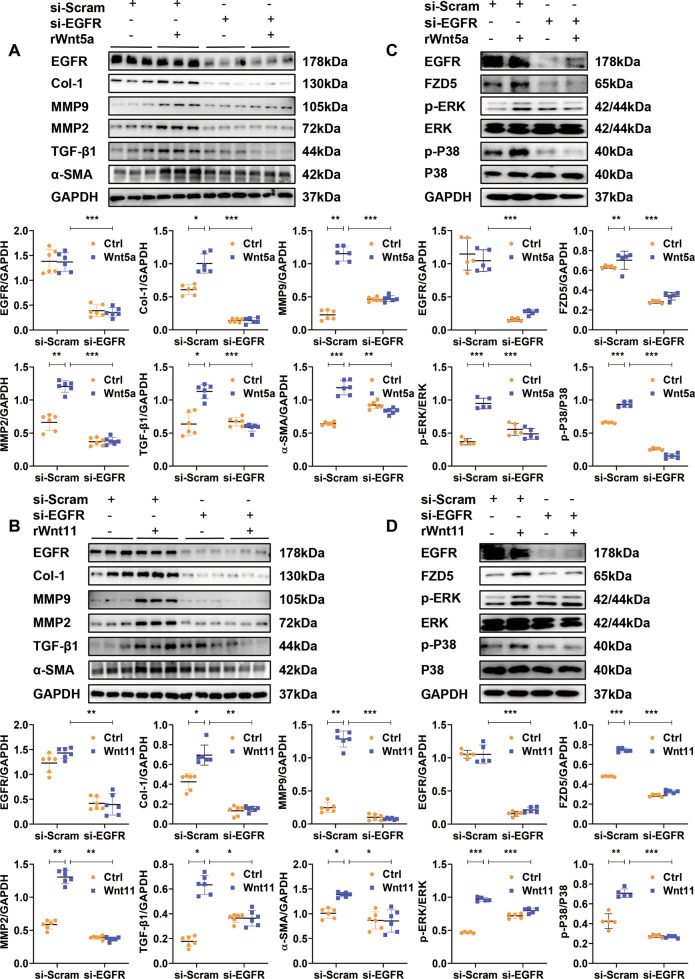
Inhibition of EGFR activation attenuates the pro-fibrotic effect induced by Wnt5a or Wnt11 in neonatal rat cardiac fibroblasts. **A**, **B** The expression of EGFR, Col-1, α-SMA, MMP9, MMP2, and TGF- β1 were detected by western blot in cardiac fibroblasts. After transfection with si-RNA-EGFR (si-EGFR) or si-RNA-scramble (si-Scram) for 48 h, these fibroblasts were treated with recombinant rat Wnt5a (rWnt5a) or Wnt11 (rWnt11) for 24 h, respectively. The same volume of PBS was treated as a control. *n* = 6/group. **C**, **D** The levels of p-ERK, p-P38, FZD5, and EGFR were examined by western blot analysis in cardiac fibroblasts. These fibroblasts were transfected with si-RNA-EGFR (si-EGFR) or si-RNA-scramble (si-Scram) for 48 h and then treated with recombinant rat Wnt5a (rWnt5a) or Wnt11 (rWnt11), respectively, 30 min later, these cells were analyzed. *n* = 5/group. All data were shown as mean ± SEM; *P < 0.05, **P < 0.01, ***P < 0.001.

EGFR were knocked down by si-EGFR transfection in CFs treated with Wnt5a or Wnt11. Si-EGFR greatly inhibited the fibrotic effects characterized by the decreased expression of Col-1, α-SMA, MMP9, MMP2, and TGF-β1 in CFs with Wnt5a or Wnt11 treatment (Fig. 7A, B). Similarly, Si-EGFR inhibited the activation of p38 and ERK signaling mediated by Wnt5a or Wnt11 in CFs (Fig. 7C, D).

FZD5 has been reported to be involved in the signaling induced by Wnt5a [13]. Wnt5a or Wnt11 greatly increased the expression of FZD5 in CFs, which was significantly attenuated by si-EGFR or EGFR inhibitor (Fig. 7C, D and Fig. S5C, D). But, in basic conditions, si-EGFR significantly suppressed the expression of FZD5 in CFs.

EGFR has been reported to be activated in CMs or CFs by MS [12]. We also found EGFR inhibitor, erlotinib, attenuated the elevated Wnt5a or Wnt11 level in conditional medium of cultured CMs or CFs under MS (Fig. S5E, F).

These data suggested that Wnt5a or Wnt11 activates CFs by EGFR signaling, and FZD5 may be involved in the effects. EGFR activation is also a participant in the increased secretion of Wnt5a or Wnt11 from CMs or CFs under MS.

### SiRNA-FZD5 attenuates the activation of neonatal rat CFs induced by Wnt5a or Wnt11

**Fig. 8:**
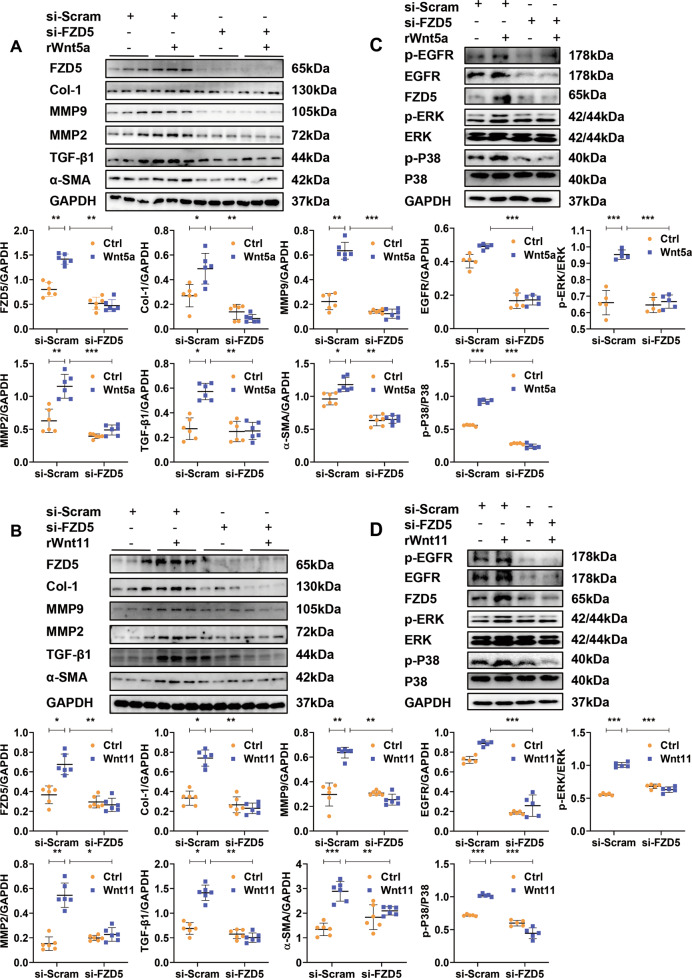
SiRNA-FZD5 attenuates the activation of neonatal rat cardiac fibroblasts induced by Wnt5a or Wnt11. **A**, **B** Western blot analysis of FZD5, Col-1, α-SMA, MMP9, MMP2, and TGF-β1 expression in cardiac fibroblasts. Si-RNA-FZD5 (si-FZD5) or si-RNA-scramble (si-Scram) was treated with fibroblasts for 48 h, then exogenous recombinant rat Wnt5a (rWnt5a) or Wnt11 (rWnt11) were added into cultured fibroblasts for 24 h for further analysis. *n* = 6/group. **C**, **D** Western blot analysis of p-EGFR, EGFR, p-ERK, p-P38, and FZD5 levels in cardiac fibroblasts. Si-RNA-FZD5 (si-FZD5) or si-RNA-scramble (si-Scram) was treated into fibroblasts for 48 h, then exogenous recombinant rat Wnt5a (rWnt5a) or Wnt11 (rWnt11) were added into cultured fibroblasts for 30 min for further analysis. *n* = 5/group. All data were shown as mean ± SEM; *P < 0.05, **P < 0.01, ***P < 0.001.

In vivo, shWnt5a/11-AAV9 pre-injection significantly attenuated the increased expression of FZD5 induced by TAC (Fig. S5A). To detect whether Wnt5a or Wnt11 activates fibroblasts by FZD5 signaling, we abolished FZD5 expression by si-FZD5 transfection in CFs with or without Wnt5a or Wnt11 treatment (Fig. 8A, B). Wnt5a or Wnt11 significantly enhanced the expression of Col-1, α-SMA, MMP9, MMP2, and TGF-β1 in fibroblasts, and the effects were significantly reversed by siFZD5 (Fig. 8A, B). P-EGFR, p-P38, and p-ERK levels showed similar results (Fig. 8C, D). Si-FZD5 also greatly decreased the expression of EGFR in fibroblasts under basic conditions.

These data indicated that Wnt5a or Wnt11 activates CFs by FZD5 signaling.

## Discussion

In the present study, we revealed that serum Wnt5a and Wnt11 were increased in patients with hypertension as compared to healthy controls. Serum Wnt5a and Wnt11 levels were positively correlated with cardiac diastolic dysfunction. During pressure overload, CFs- or CMs-secreted-Wnt5a/11 contribute to reactive cardiac fibrosis by the crosstalk of FZD5 and EGFR signaling, which may lead to subsequent cardiac dysfunction (Fig. S6). The present study illustrated a novel mechanism by which cardiac fibrosis is initiated by Wnt5a or Wnt11 at the early phase of the hypertensive heart.

Wnt5a has been reported to be elevated in the serum or myocardium of heart failure patients, and the level is positively related to cardiac dysfunction [7, 8]. The mRNA level of Wnt5a is upregulated in heart tissue following MI, and overexpression of Wnt5a promotes CFs differentiation in vitro [14]. We observed Wnt5a was decreased while Wnt11 didn’t change at any time-points following MI (the data not shown). The increased serum or cardiac Wnt5a or Wnt11, derived from CMs, CFs, or CMECs, is associated with cardiac diastolic dysfunction of hypertensive patients. Abraityte A reported that the main cellular source of Wnt5a in mouse heart is CFs, they only detected the mRNA level of Wnt5a, not protein level [7]. Wnt11, an underexplored member of the Wnt family, is required for cardiogenesis or myocardium development [15, 16]. Wnt11 derived from mesenchymal stem cell protects CMs against hypoxia [17] or promotes angiogenesis during acute MI [18]. Wnt11-AAV9-injection improves cardiac function following MI by inhibition of inflammatory response [19]. Few studies reported that whether CM or CF could secret Wnt11 under pressure overload. We observed the increased secretion of Wnt5a or Wnt11 from CMs or CFs promoted cardiac fibrosis under pressure overload, which may explain the increased Wnt5a or Wnt11 level was positively associated with cardiac diastolic dysfunction. Our data also suggested that the role of Wnt11 in the heart is dependent on the type of pathological stimuli.

Wnt5a and Wnt11 are predominantly β-catenin-independent Wnts [20]. Wnt5a induces IL-6 and TIMP-1 by activation of ERK in CFs [7], resulting in cardiac inflammation and subsequent heart failure progression. Wnt5a promotes TGFβ1-mediated macrophage polarization by stimulating Yap/Taz during kidney fibrosis [21]. Wnt11 mediates the actions of TGF-β in primary renal epithelial cells by enhancing mesenchymal marker genes such as α-SMA [11], which promotes renal interstitial fibrosis. Wnt11 enhances osteogenic differentiation by activation of p38 signaling [22]. In this study, Wnt5a or Wnt11 activated P38 and ERK pathway, which are involved in the development of cardiac fibrosis [12], to exert the pro-fibrotic effects.

The activation of EGFR has been involved in cardiac fibrosis under pressure overload [12]. Crosstalk between Wnt and EGFR has been identified in many tumors including breast cancers [23, 24]. In HC11 mammary epithelial cells, Wnt1 and Wnt5a induced the activation of EGFR and MAPK [25]. Our data indicated that Wnt5a or Wnt11 activated EGFR by FZD5 to promote cardiac fibrosis under pressure overload. FZD receptors, belonging to the G protein-coupled receptor superfamily, bind multiple Wnt molecules with an extracellular cysteine-rich domain to stimulate diverse signaling cascades [20]. EGFR has been reported to be trans-activated by G protein-coupled receptors independent or dependent of EGF-like ligands [26, 27].

Wnt5a interacts with various members of the FZD family (FZD2, 3, 4, 5, 7, and 8) as well as the other co-receptors such as LRP5/6 [13, 20]. FZD5 has been identified to be a preferred receptor of Wnt5a, which mediates the antiproliferative and proapoptotic effects in prostate cancer [13], and inflammatory reactions in sepsis [28]. But few studies reported the interaction of Wnt11 and FZD5 involved in biological function. Our data revealed that Wnt5a or Wnt11 activates FZD5 to promote cardiac fibrosis under pressure overload. It suggested that the activation of the FZD receptor by Wnt5a or Wnt11 is cell or tissue-specific, or dependent on the type of pathological stimuli. We also observed EGFR expression is regulated by FZD5 in CFs under basic conditions. In hematopoietic stem cells, EGFR is required as a cofactor for Wnt9a–Fzd9b signaling [29]. So it also is possible that Wnt5a or Wnt11 recruits EGFR, leading to its regulation of FZD5 intracellular domains to mediate cardiac fibrosis.

In addition to Wnt5a or Wnt11, other pro-fibrotic molecules may be induced by pressure overload. We previously reported CMs-secretory β2M or microRNA-378 promotes cardiac fibrosis under pressure overload [12, 30]. Considering the adaptive response in vivo, we did not knockdown of Wnt5a or Wnt11 selectively in CMs or CFs to observe the pro-fibrotic effect under pressure overload. Whether Wnt5a or Wnt11 causes the direct interaction of FZD5 and EGFR needs future study. The limitation of the study is that we used neonatal CFs not adult fibroblasts to explore the potential mechanisms.

In conclusion, pressure overload-induced the increased Wnt5a or Wnt11 expression and secretion from CMs or CFs, which promotes cardiac fibrosis by crosstalk of FZD5 and EGFR signaling. It may explain, in our clinical observation, the elevated serum Wnt5a or Wnt11 level and positively corrected with cardiac diastolic dysfunction in hypertension patients. The current study indicated the importance of Wnt5a and Wnt11 as pro-fibrotic mediator. Inhibition of Wnt5a or Wnt11 may have therapeutic potential for the prevention of cardiac fibrosis associated with hypertension or pressure overload.

## Supplementary information

Supplementary material

Fig.S1

Fig.S2

Fig.S3

Fig.S4

Fig.S5

Fig.S6

## Data Availability

The data analyzed during the current study are available from the corresponding author on reasonable request.
