# Supplementary material for: Cardiac Wnt5a and Wnt11 promote fibrosis by the crosstalk of FZD5 and EGFR signaling under pressure overload
Source: Cell Death Dis. 2021 Sep 25;12(10):877. doi: 10.1038/s41419-021-04152-2 (PMC8464604; doi:10.1038/s41419-021-04152-2)
Supplement: Supplementary file 1 — Supplementary material [file 41419_2021_4152_MOESM1_ESM.docx]

**Supplementary material**

Table S1. The primers used in real-time PCR analysis

| Gene name | Forward primer, 5’→3’ | Reverse Primer, 3’→5’ |
| --- | --- | --- |
| ANP | GGTGTCCAACACAGATCTGA | CCACTAGACCACTCATCTAC |
| BNP | AAGCTGCTGGAGCTGATAAGA | GTTACAGCCCAAACGACTGAC |
| Wnt5a | GAGACAACATCGACTATGGCTA | CAGGTTGTATACTGTCCTACGG |
| Wnt11 | AATCAGACGCAACACTGTAAAC | CTCGATGGAGGAGCAGTTC |
| Wnt1 | GGGTTTCTACTACGTTGCTACT | CAGACTCTTGGAATCCGTCAA |
| Wnt4 | AGTTCAAGCCACATACAGATGA | TTTAGATGTCTTGTTGCACGTG |
| Wnt7a | TGCCTTCACCTATGCGATTATC | CGCCTCGTTATTGTGTAAGTTC |
| Wnt10b | GAGAAGTTCTCTCGGGATTTCT | CTTCAGGTTTTCCGTTACCAC |

Table S2.Baseline characteristics in healthy subjects group (Control), patients with hypertension (HBP)

| **Variable** | **Control**  **(n=33)** | **HBP**  **(n=56)** | **P Value** |
| --- | --- | --- | --- |
| Male | 21 (63.6%) | 36 (64.3%) | 0.857 |
| Age,years | 62 ± 9 | 62 ± 7 | 0.154 |
| SBP (mmHg) | 127 ± 11 | 137 ± 18 | 0.012 |
| DBP (mmHg) | 74 ± 9 | 81 ± 10 | 0.001 |
| Heart rate (bpm) | 69 ± 6 | 72 ± 10 | 0.177 |
| **Echocardiograph data** | | | |
| LVEDD (mm) | 47.00±3.70 | 48.49±6.44 | 0.181 |
| LVESD (mm) | 30.19±3.04 | 31.70±6.46 | 0.138 |
| EF (%) | 63.74±6.51 | 62.92±8.49 | 0.324 |
| Septal thickness (mm) | 9.68±1.40 | 10.24±1.60 | 0.116 |
| Posterior wall thickness (mm) | 9.28±0.99 | 9.54±1.10 | 0.367 |
| E (cm/s) | 70.05 ± 15.56 | 76.68 ± 16.66 | 0.047 |
| e’ (cm/s) | 8.87 ± 2.47 | 6.85 ± 2.85 | 0.002 |
| E/e’ ratio | 8.41 ± 2.88 | 13.31 ± 6.73 | <0.001 |
| **Laboratory data** | | | |
| Creatinine (μmol/L) | 75.36 ± 17.10 | 79.11±20.01 | 0.547 |
| ALT (U/L) | 25.02±12.75 | 23.14 ± 9.16 | 0.339 |
| AST (U/L) | 20.33±7.34 | 19.21 ± 5.65 | 0.308 |
| TC (mmol/L) | 3.97±0.87 | 3.91 ± 0.97 | 0.865 |
| TG (mmol/L) | 1.77±1.02 | 1.98 ± 0.92 | 0.355 |
| BNP (pg/ml) | 213.91 ± 69.39 | 451.95 ± 151.84 | 0.291 |
| CK (U/L) | 80.50 ± 40.34 | 103.78 ± 74.21 | 0.317 |
| Lpa (mg/L) | 278.93 ± 71.57 | 208.40 ± 61.11 | 0.241 |
| ApoE (mg/dl) | 40.51±16.80 | 42.44±16.21 | 0.849 |
| **Medical history** | | | |
| Smoke | 11 (33.3%) | 23 (41.1%) | 0.549 |
| Diabetes | 10 (30.3%) | 25 (44.6%) | 0.228 |

*Data are expressed as mean±SD; SBP:systolic blood pressure; DBP:diastolic blood pressure; LVEDD:left ventricular end diastolic diameter; LVESD:left ventricular end systolic diameter; EF:ejection fraction; ALT:alanine aminotransferase; AST:aspartate aminotransferase; TC:total cholesterol; TG:total triglyceride; BNP:brain natriuretic peptide; CK:creatine kinase; Lpa:Lipoprotein(a); ApoE:apolipoprotein E.

Table S3. The relationship between serum Wnt5a/Wnt11 and echocardiographic parameters of healthy subjects (Control) and patients with hypertension (HBP)

| **Parameter** | **serum** | **Correlation(r)** | **P Value** |
| --- | --- | --- | --- |
| LVEDD (mm) | Wnt5a | 0.127 | 0.234 |
|  | Wnt11 | 0.061 | 0.571 |
| LVESD (mm) | Wnt5a | 0.133 | 0.210 |
|  | Wnt11 | 0.07 | 0.514 |
| EF (%) | Wnt5a | -0.148 | 0.163 |
|  | Wnt11 | 0.360 | 0.737 |
| Septal thickness (mm) | Wnt5a | 0.166 | 0.119 |
|  | Wnt11 | 0.113 | 0.291 |
| Posterior wall thickness (mm) | Wnt5a | 0.071 | 0.507 |
|  | Wnt11 | -0.079 | 0.457 |
| E (cm/s) | Wnt5a | 0.102 | 0.334 |
|  | Wnt11 | 0.113 | 0.286 |
| e’ (cm/s) | Wnt5a | -0.217 | 0.039 |
|  | Wnt11 | -0.259 | <0.001 |
| E/e’ ratio | Wnt5a | 0.273 | 0.009 |
|  | Wnt11 | 0.32 | 0.003 |

LVEDD: left ventricular end diastolic diameter; LVESD: left ventricular end systolic diameter; EF: left ventricular ejection fraction.

Table S4. The relationship between serum Wnt5a/Wnt11 and echocardiographic parameters (E, e’ and E/e’) of the participants independent of the factors including smoke and diabetes.

| **Parameter** | **serum** | **Correlation(r)** | **P Value** |
| --- | --- | --- | --- |
| E (cm/s) | Wnt5a | 0.150 | 0.462 |
|  | Wnt11 | 0.151 | 0.417 |
| e’ (cm/s) | Wnt5a | -0.387 | 0.031 |
|  | Wnt11 | -0.577 | 0.001 |
| E/e’ ratio | Wnt5a | 0.455 | 0.010 |
|  | Wnt11 | 0.480 | 0.006 |

**Fig S1. Pressure overload induces the increased expression of cardiac fibrosis.**

1. . The expressions of Col-1, MMP9, MMP2, TGF-β1 and α-SMA were analyzed by western blot in the heart tissues of patients with dilated cardiomyopathy(DCM) or control group, n=4/group. (B).Schematic diagram of operation time points in sham or TAC mice. (C).Western blot analysis of Col-1, MMP9, MMP2, TGF-β1 expressions in heart tissues from TAC mice at different time-points (3d, 1w, 2w, 4w), n=4/group.All data are shown as mean±SEM; * P< 0.05, ** P<0.01, *** P<0.001.

**Fig S2. Expression of Wnt5a and Wnt11 in cardiac cells under pressure overload.**

(A).The immunofluorescence staining of neonatal rat cardiomyocytes with α-MHC antibody (Red). Bar=20μm. (B).The expressions of Wnt5a and Wnt11 in cardiomyocytes and fibroblasts were analyzed by Western blot analysis under basic condition. n=6/group. (C).Schematic diagram of time points after mechanical stretch (MS). (D). Western blot analysis of Wnt5a and Wnt11 expression in α-SMA positive or negative fibroblasts. α-SMA positive or negative fibroblasts were sorted by FACS. (E).The expressions of Wnt5a and Wnt11 in neonatal cardiac microvascular endothelial cells (CMECs) were analyzed by Western blot analysis in stretched (MS) or control (Ctrl) condition. n=3/group. All data are shown as mean±SEM; * P< 0.05, ** P<0.01, *** P<0.001.

**Fig S3. Wnt5a or Wnt11 contributes to cardiac fibrosis and hypertrophy induced by pressure overload.**

Mice were injected with sh-Wnt5a /Wnt11-AAV9 (sh-Wnt5a /Wnt11) or sh-scramble-AAV9 (sh-NC) by tail vein, 2 weeks later, TAC or sham operation was used to induce cardiac pressure overload in mice. After 4 weeks, further analysis was performed. (A). Real-time PCR analysis of Wnt5a, Wnt11, Wnt1, Wnt4, Wnt7a, Wnt10b mRNA levels of TAC mice pre-injected with sh-Wnt5a/Wnt11 or sh-NC. n=4/group. (B). Echocardiographic analysis of FS (shortening fraction) in sham or TAC mice pre-injected with sh-Wnt5a/Wnt11 or sh-NC. n=5/group; TAC: n=4/group. (C, D). HW/BW (heart weight/body weight) ratio and CSA (cross sectional area) of cardiomyocytes were analyzed. n=6 or 11/group. (E).The mRNA expression levels of ANP and BNP were analyzed by real-time PCR. n=5/group. (F). Western blot analysis of Col-1, MMP9 and MMP2 expressions in heart tissue. Mice were infused with rat recombinant Wnt5a and Wnt11 proteins via minipump, and then subjected with TAC or sham operation. After 4 weeks, further analysis was performed. n=4/group. (G). HW/BW (heart weight/body weight) ratio was analyzed in mice as (F). n=6/group. (H) Echocardiographic analysis of FS (shortening fraction) in mice as (F). n=6/group. All data are shown as mean±SEM; * P< 0.05, ** P<0.01, *** P<0.001.

**Fig S4. Exogenous rat Wnt5a and Wnt11 induces neonatal rat cardiac fibrosis and hypertrophy.**

(A, B). Western blot was used to analyze the expression level of Col-1, MMP9, MMP2, TGF-β1, p-Smad2/3 and α-SMA in fibroblasts stimulated by rWnt5a (50ng/ml) or rWnt11 (50ng/ml), n=3/group. (C). Western blot analysis of p-ERK and ERK level in cultured neonatal rat cardiaomyocytes treated with recombinant Wnt5a or Wnt11. n=3/group. All data are shown as mean±SEM; * P< 0.05, ** P<0.01, *** P<0.001.

**Fig S5. EGFR-specific inhibitors (Erlotinib) effectively inhibits the fibrosis in neonatal rat cardiac fibroblasts treated with Wnt5a or Wnt11.** (A).The expression levels of EGFR, p-EGFR and FZD5 were analyzed by Western blot in heart tissue from sham or TAC mice pre-injected with sh-Wnt5a /Wnt11-AAV9 (sh-Wnt5a /Wnt11) or sh-scramble-AAV9 (sh-NC). n=6/group. (B). ELISA analysis of Wnt5a and Wnt11 levels in the supernatant of fibroblasts under MS or Wnt5a and Wnt11 recombinant proteins stimulation. n=6/group. (C, D). The expressions of Col-1, MMP9, MMP2 and TGF-β1 was detected in cardiac fibroblasts. These fibroblasts was stimulated with Erlotinib (2 μM), an EGFR inhibitor for 1h and then incubated with exogenous recombinant proteins Wnt5a or Wnt11 for 24h .n=6/group. (E, F) ELISA analysis of the concentration of Wnt5a or Wnt11 in conditional medium from stretched (MS) or control cardiomyocytes (CMs) or cultured cardiac fibroblasts (CFs) pretreated with Erlotinib (2 μM). n=6/group. All data are shown as mean±SEM; * P< 0.05, ** P<0.01, *** P<0.001.

**Fig S6. The summary of the present study.**

Under pressure overload, CFs- or CMs-secreted-Wnt5a/11 contribute to reactive cardiac fibrosis by the crosstalk of FZD5 and EGFR signaling, which may lead to subsequent cardiac dysfunction in hypertensive patients.
