# Supplementary figures and images for: Cardiac Wnt5a and Wnt11 promote fibrosis by the crosstalk of FZD5 and EGFR signaling under pressure overload
Source: Cell Death Dis. 2021 Sep 25;12(10):877. doi: 10.1038/s41419-021-04152-2 (PMC8464604; doi:10.1038/s41419-021-04152-2)

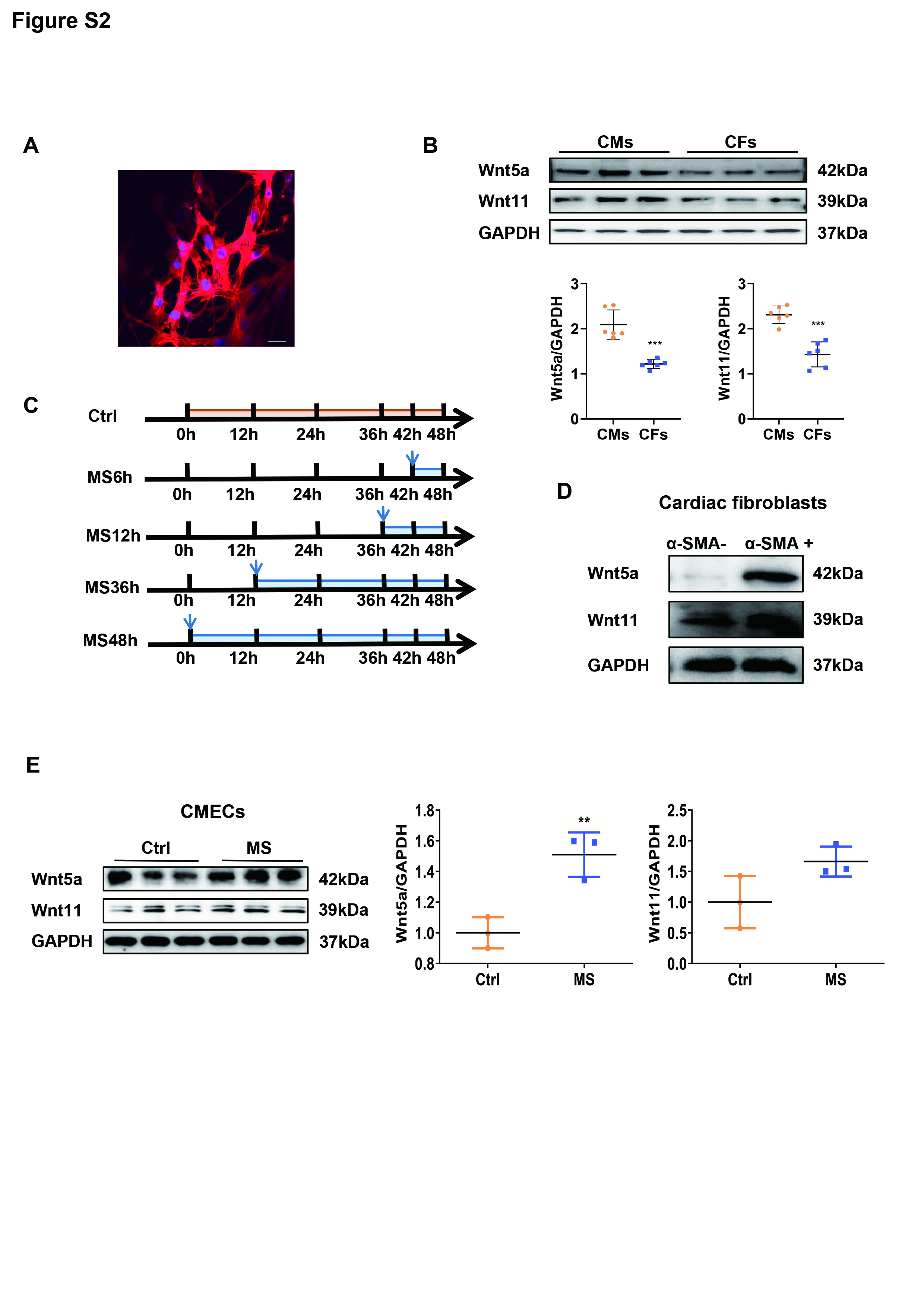

Supplement: Supplementary file 3 — Fig.S2 [file 41419_2021_4152_MOESM3_ESM.tif]

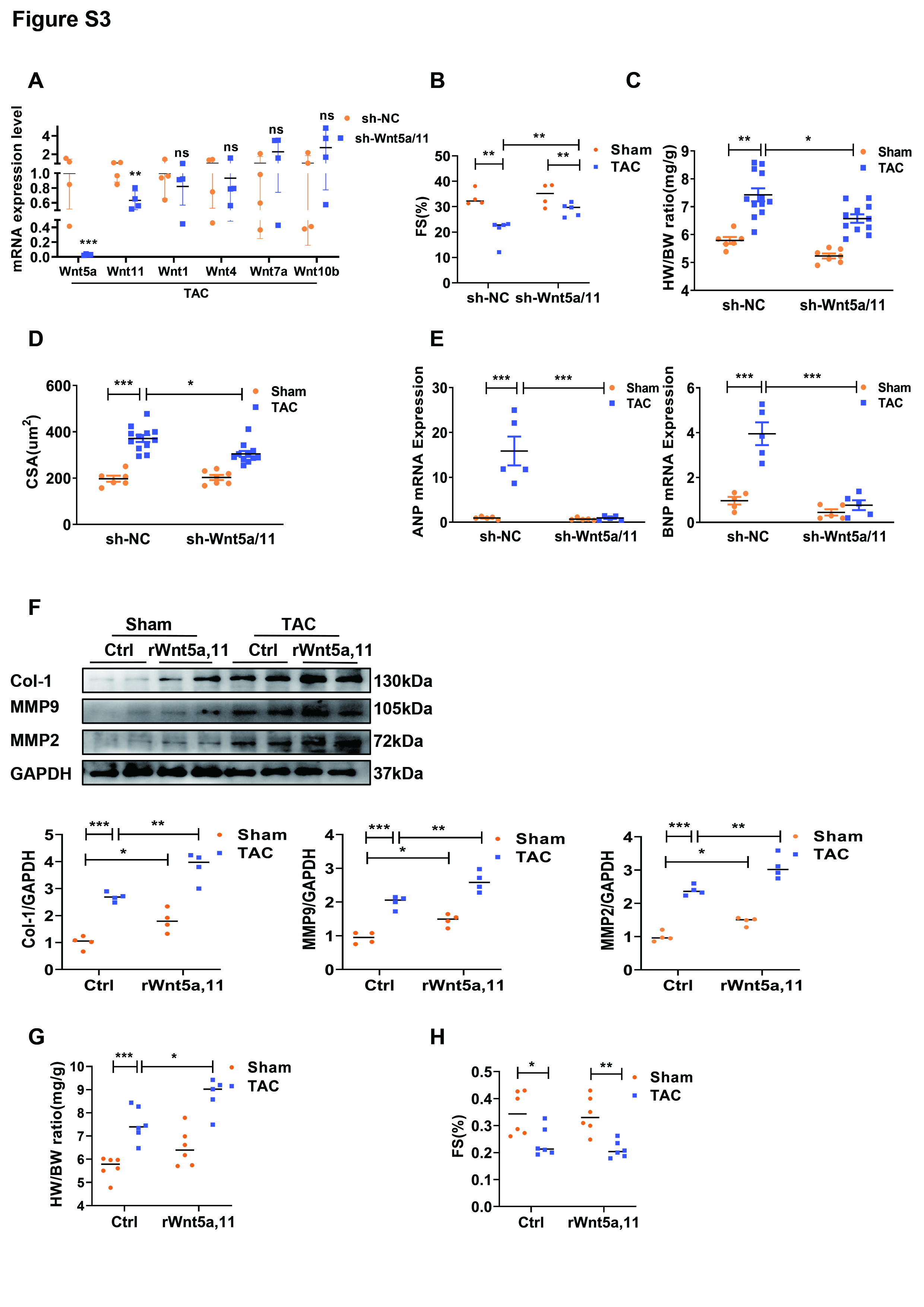

Supplement: Supplementary file 4 — Fig.S3 [file 41419_2021_4152_MOESM4_ESM.tif]

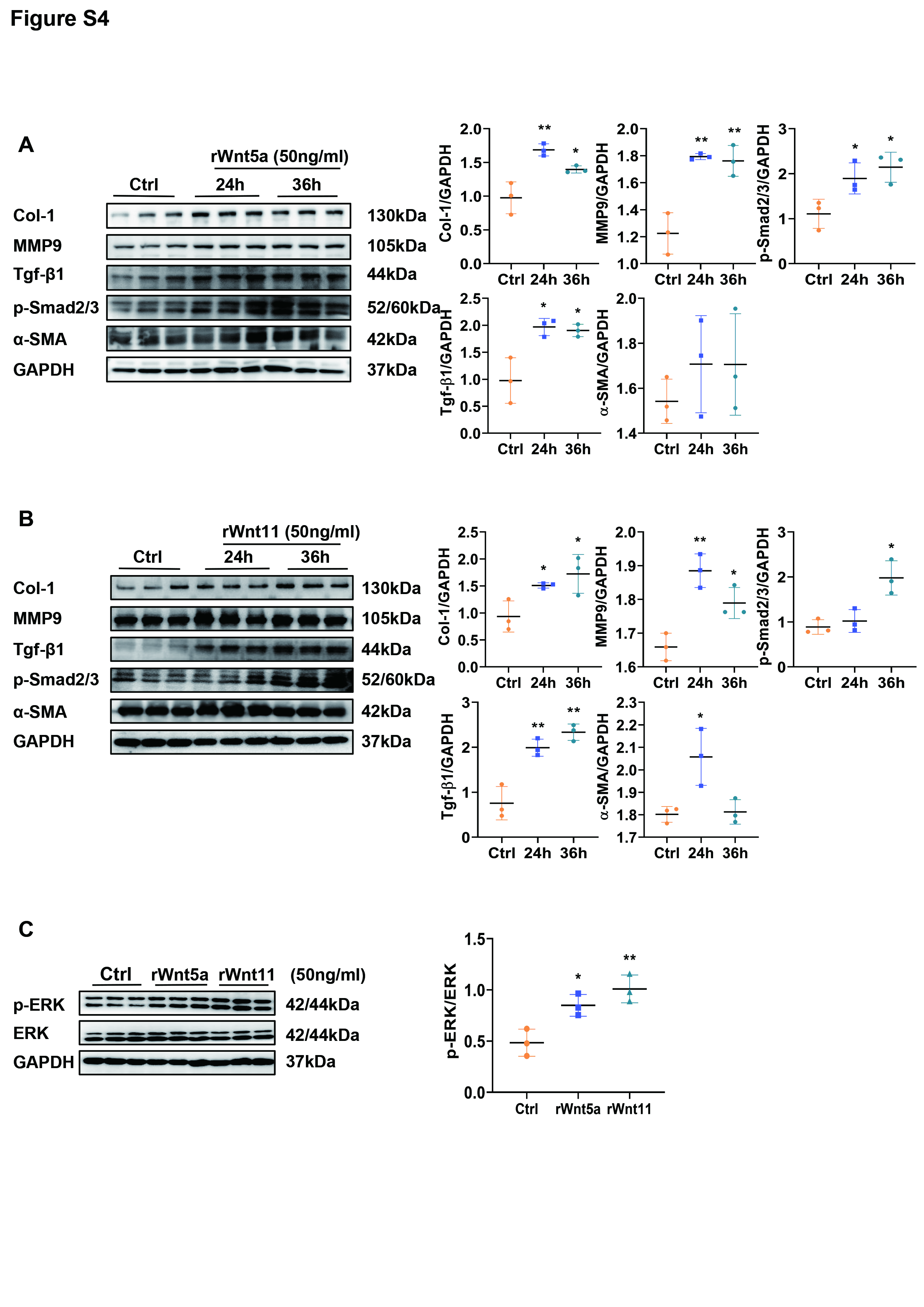

Supplement: Supplementary file 5 — Fig.S4 [file 41419_2021_4152_MOESM5_ESM.tif]

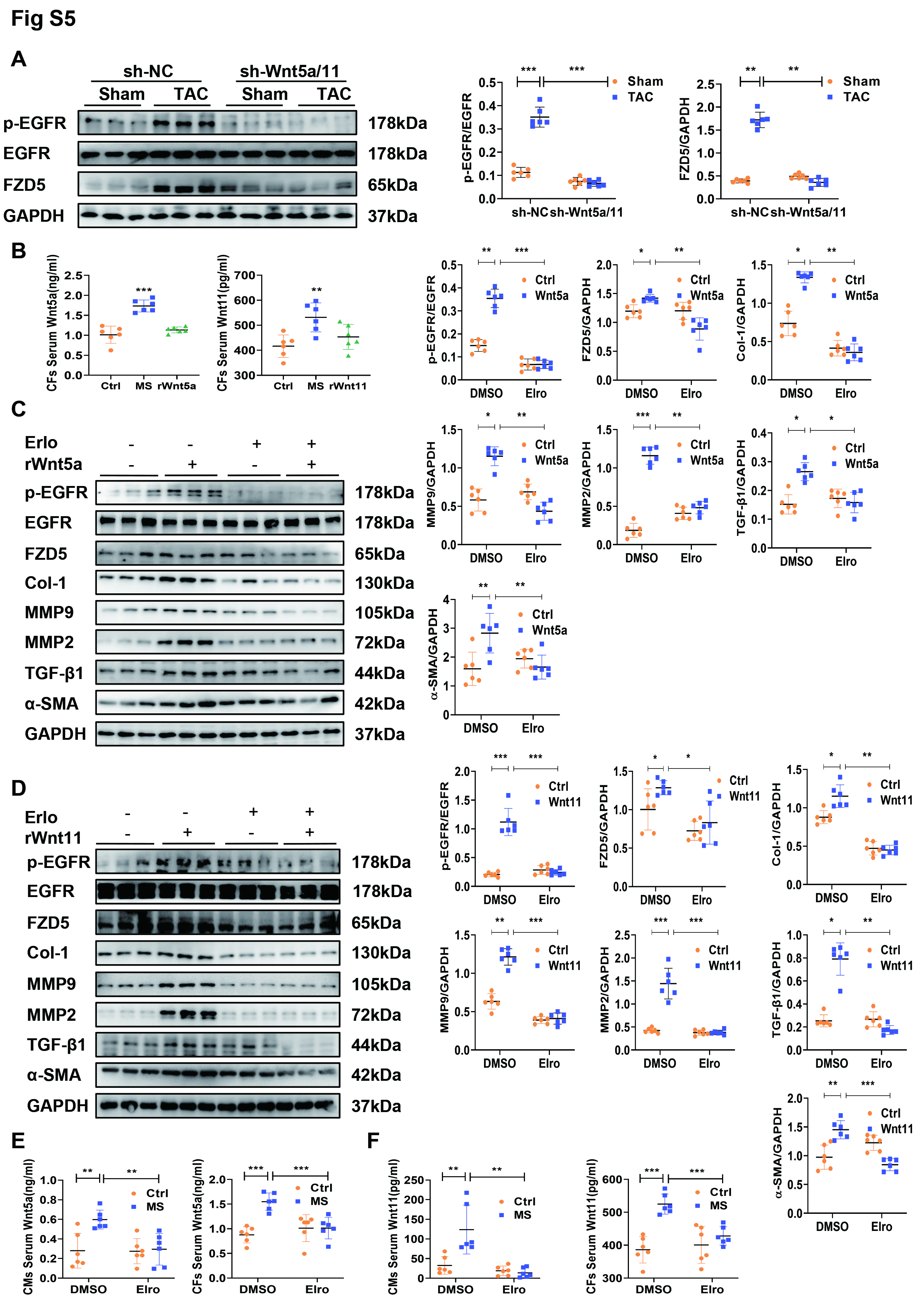

Supplement: Supplementary file 6 — Fig.S5 [file 41419_2021_4152_MOESM6_ESM.tif]
